# Extracellular Molecular Repertoire of Xerotolerant Actinobacteria Colonizing Serpentinite Rocks

**DOI:** 10.3390/ijms27104233

**Published:** 2026-05-09

**Authors:** Anna A. Elistratova, Elizaveta N. Dekhanova, Dilyara R. Kamaldinova, Elena I. Shagimardanova, Margarita R. Sharipova, Michael F. Cohen, Irina V. Khilyas

**Affiliations:** 1 Institute of Fundamental Medicine and Biology, Kazan (Volga Region) Federal University, 420008 Kazan, Russia; 2 Laboratory of Multiomics Technologies of Living Systems, Institute of Fundamental Medicine and Biology, Kazan (Volga Region) Federal University, 420008 Kazan, Russia; 3 Genomic and Bioimaging Core Facility, 95112 Moscow, Russia; 4 Life Improvement by Future Technologies (LIFT) Center, 121205 Moscow, Russia; 5 University of California Cooperative Extension, Santa Clara County, San Jose, CA 95112, USA

**Keywords:** bacterial stress response, osmoadaptation, compatible solutes, *Actinomycetota*, nonribosomal peptide synthetases (NRPS), surface-active compounds, endolithic, ultramafic rocks, heavy metal resistance, extreme environments

## Abstract

Weathered serpentinites are extreme lithobiontic environments characterized by oligotrophy, high heavy metal content, and desiccation stress; yet, the adaptive mechanisms of colonizing actinobacteria remain poorly understood. This study aimed to isolate and characterize xerotolerant actinobacteria from serpentinite and to profile their secondary metabolites involved in stress tolerance. Three lithobiontic strains were isolated and identified by whole-genome sequencing (dDDH and ANI) as *Rhodococcus oxybenzonivorans* SK11, *Paenarthrobacter nitroguajacolicus* SK18, and *Rhodococcus qingshengii* SK25. Desiccation tolerance was assessed using PEG-8000, siderophore production on CAS agar with metal substitution (Fe^3+^, Al^3+^, Cu^2+^, Ga^3+^), and biosurfactant activity via emulsification assays. Genome mining identified biosynthetic gene clusters for compatible solutes, siderophores, and biosurfactants. All strains maintained viability at 50% PEG. Compatible solute pathways included ectoine (*ectABC*) in SK18 and SK25, glycine betaine (*gbsAB*) only in SK18, and trehalose (TreYZ) and proline (ProABC) pathways in all three. Genome mining of *Rhodococcus* strains revealed a number of NRPS-dependent clusters, some of which are predicted to encode siderophores (rhodochelin, heterobactins), while SK18 used an NRPS-independent desferrioxamine E pathway together with a unique lanthipeptide cluster. Biosurfactant production was condition-dependent, with SK25 achieving complete emulsification (E_24_ = 100%) in hexadecane-supplemented medium. These findings demonstrate that weathered serpentinite actinobacteria employ an extracellular molecular repertoire of compatible solutes, siderophores, and biosurfactants to survive extreme oligotrophy, desiccation, and metal stress.

## 1. Introduction

Serpentinites are ultramafic rocks formed through the geochemical process of serpentinization. These rocks are primarily composed of Mg-rich silicate minerals, such as antigorite and lizardite (Mg_3_Si_2_O_5_(OH)_4_), and minor minerals including brucite (Mg(OH)_2_), talc (Mg_3_Si_4_O_10_(OH)_2_), carbonates (calcite, magnesite), and magnetite (Fe_3_O_4_) [1]. Serpentinites occur in sites of active serpentinization or as weathered formations where the process has completely finished [2,3]. The physicochemical properties of these minerals constitute extreme environments to which extremophilic microorganisms with unique metabolic systems are adapted [4,5].

Endolithic bacteria are adapted to survive within lithic environments of rocks, cracks and crevices. A key adaptive strategy for endolithic bacteria is the secretion of secondary metabolites—low-molecular weight extracellular compounds that provide protection, facilitate acclimatization, and enhance nutrient acquisition [6]. These metabolites include surface-active compounds (biosurfactants) and siderophores. Along with their biological roles, bacterial metabolites actively contribute to mineral weathering, promotion of mineral dissolution and metal solubilization [7].

Members of the phylum Actinomycetota are frequently detected in lithic-associated habitats, particularly within weathered serpentinites [8]. A distinctive feature of the strains belonging to this phylum is their high adaptability to desiccation (xerotolerance), which is attributed to the production of specialized metabolites maintaining cellular functions under low-water conditions. In particular, desiccation-tolerant actinobacteria of the class *Actinomycetes* belonging to the genera *Rhodococcus* and *Arthrobacter*—the latter of which recently underwent significant taxonomic revision into several new genera including *Paenarthrobacter* [9]—are known for their metabolic versatility and capacity to produce osmolytes, siderophores, and biosurfactants [10,11].

The mechanisms of desiccation resistance in xerotolerant bacteria, including actinobacteria, are attributed to the synthesis of compatible solutes (choline, glycine, betaine, sarcosine, proline, trehalose and ectoine) that stabilize proteins and cell membranes [12]. Actinobacteria also produce biosurfactants primarily consisting of glycolipids (trehalose lipids and rhamnolipids), phospholipids, and lipopeptides that facilitate cell adhesion to mineral surfaces and protect against desiccation [13]. Metal acquisition under harsh oligotrophic conditions is generally facilitated by secretion of hydroxamate-, catechol- and citrate-types of siderophores [14]. Biosurfactants enhance access of siderophores to surfaces by acting as wetting agents and dispersing hydrophobic coatings [15]. In addition, biosurfactants promote the initial mobilization of mineral-bound iron into the aqueous phase [16], increasing the availability of iron for subsequent chelation by higher-affinity siderophores. Overlapping global regulatory networks (including quorum sensing, iron-responsive regulators such as Fur, and stress-response systems) can lead to condition-dependent co-expression of biosurfactants and siderophores [17,18,19].

Therefore, the objective of this study was to isolate and characterize the phylogenetic diversity and metabolic potential of xerotolerant lithobiontic actinobacteria from weathered serpentinites. Our primary goal was profiling their repertoire of secondary metabolites including siderophores and biosurfactants that contribute to desiccation tolerance and facilitate survival in lithobiontic conditions.

## 2. Results

### 2.1. Isolation and Identification of the Lithobiontic Strains

All isolates were Gram-positive and non-sporulating rod-shaped cells (0.5–0.7 μm wide, 2–5 μm long). They formed small (0.5–1.0 mm), round colonies with a smooth, glistening surface: pale orange for SK11, or milky-white for SK25, bright yellow for SK18 after 72 h of growth on LA at 30 °C (Figure S1).

Subsequent whole-genome sequencing and analysis provided a more comprehensive genomic characterization of the isolates. The draft genome assemblies revealed the following genome sizes: SK11 − 7,821,393 bp, SK18 − 4,762,147 bp, and SK25 − 6,886,367 bp. The DNA G+C content was determined to be 65% for SK11, and 62% for both SK18 and SK25 (Table S2). Digital DNA−DNA hybridization (dDDH) and average nucleotide identity (ANI) analyses confirmed the species-level identification for all three strains. SK11 exhibited a dDDH value of 72.4% (confidence interval 69.4–75.3%) with the type strain *Rhodococcus oxybenzonivorans* S2-17 and ANI of 98.6% with *R. oxybenzonivorans* IEGM 28, well above the 70% and 95–96% thresholds for prokaryotic species delineation, respectively (Table S3). SK18 showed a dDDH value of 79.1% (76.1–81.8%) with the type strain *Paenarthrobacter nitroguajacolicus* JCM 14115 and ANI of 98.5% with *P. nitroguajacolicus* G2-1 (Table S4). SK25 demonstrated dDDH values of 87.4% (84.8–89.5%) with *Rhodococcus qingshengii* JCM 15477 and 88.6% (86.2–90.7%) with *Rhodococcus jialingiae* djl-6-2 (a homotypic synonym of *R. qingshengii*), with an ANI of 98.7% to *R. qingshengii* djl-6-2 (Table S5). Consistent with these genomic results, MALDI-TOF MS analysis (Biotyper) using direct colony transfer identified strain SK18 as *Paenarthrobacter nitroguajacolicus* and found strains S11 and S25 to have closest matches to *Rhodococcus* species (Table S1).

**Figure 1:**
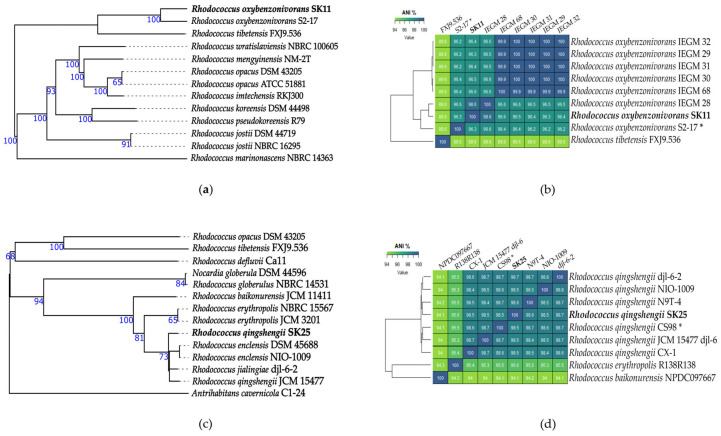

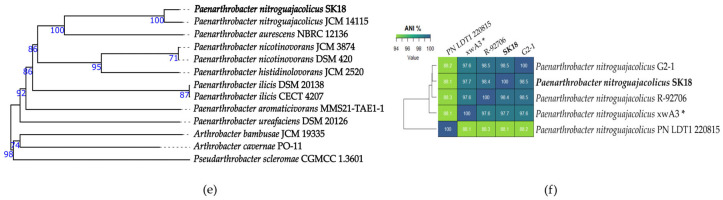
(**a**–**c**) Phylogenomic placement and genomic relatedness of lithobiontic isolates SK11, SK18, and SK25 within the genera *Rhodococcus* and *Paenarthrobacter*; (**d**–**f**) Genome BLAST distance phylogeny (GBDP) trees based on in silico DNA−DNA hybridization (DDH) comparisons. Branch lengths are scaled according to GBDP distance formula d5. Numbers above branches indicate GBDP pseudo-bootstrap support values >60% (100 replications), with average branch supports of 94.9%, 88.8%, and 68.2% for panels (**a**), (**b**), and (**c**), respectively. Heat maps displaying pairwise average nucleotide identity (ANI) values among selected *Rhodococcus* and *Paenarthrobacter* genomes, confirming the taxonomic assignments of strains SK11, SK18, and SK25. Asterisks indicate type strains.

Based on these results, the taxonomic identities of SK11, SK18, and SK25 were determined to be *Rhodococcus oxybenzonivorans*, *Paenarthrobacter nitroguajacolicus*, and *Rhodococcus qingshengii*, respectively (Figure 1).

### 2.2. Phenotypic Characterization

**Figure 2:**
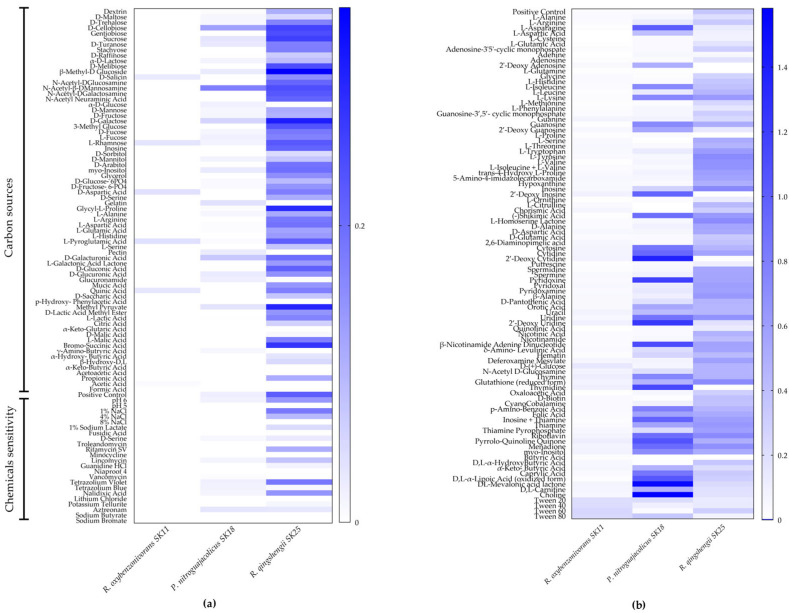
Phenotypic profiling of lithobiontic actinobacterial strains. Heat maps showing the metabolic activity of the studied strains based on (**a**) BIOLOG Gen III Microplate and (**b**) BIOLOG PM5 MicroPlate analyses. Respiration of a standardized cell suspension was measured using a tetrazolium-based redox dye, where increased metabolic activity results in higher color intensity formation. The values represent the mean absorbance readings after 24 h of incubation.

All lithobiontic strains were characterized using Biolog GEN III microplates testing for carbon sources, polymers utilization, amino acid metabolism, antibiotic/antiseptic sensitivity, and growth across pH and salinity gradients (Figure 2). Strains showed different substrate utilization profiles, with *R. qingshengii* SK25 demonstrating the broadest metabolic versatility. PM5 microplate assays revealed strain-specific differences in biosynthetic pathway and nutrient stimulation responses. *R. oxybenzonivorans* SK11 was distinguished by its significantly weaker substrate utilization and limited capabilities compared to all other strains (Figure 2).

### 2.3. Survival of Lithobiontic Strains Under Desiccation Stress

**Figure 3:**
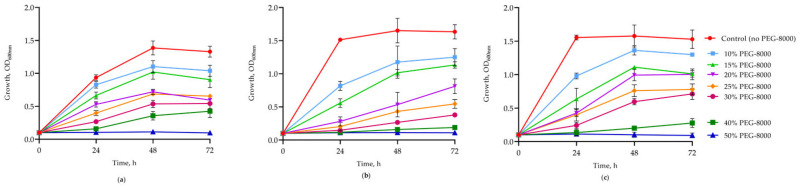
Growth of lithobiontic actinobacterial strains (**a**), *R. oxybenzonivorans* SK11; (**b**), P. *nitroguajacolicus* SK18; (**c**), *R. qingshengii* SK25 in Mueller–Hinton broth supplemented with different concentrations of PEG-8000. Data represent mean ± SD from two independent experiments.

**Figure 4:**
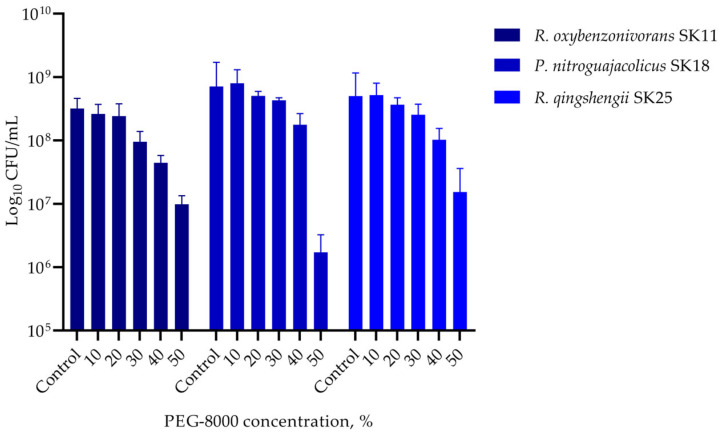
Viability of the three strains after 7 days of exposure to growth medium supplemented with PEG-8000. Survival of cells assayed turbiometrically for growth in Figure 3 was determined by colony-forming unit (CFU/mL) enumeration on LA agar plates. Data represent mean ± SD from two independent experiments.

To assess the desiccation tolerance, all lithobiontic strains were cultivated in Mueller–Hinton broth supplemented with increased concentrations (0–50%) of PEG-8000 to induce water stress, and growth assessed turbiometrically. *R. qingshengii* SK25 and *R. oxybenzonivorans* SK11 showed the highest resistance, displaying growth in up to 40% PEG, while the xerotolerance of *P. nitroguajacolicus* SK18 was lower (Figure 3). All three strains maintained viability at PEG-8000 concentrations as high as 50%, as determined by CFU enumeration (Figure 4).

Genome mining revealed distinct capacities for compatible solute biosynthesis among the three lithobiontic actinobacterial strains (Table 1 and Table S6). *P. nitroguajacolicus* SK18 and *R. qingshengii* SK25 possess complete *ectABC* operons for ectoine biosynthesis, whereas *R. oxybenzonivorans* SK11 lacks the ectoine synthase gene, indicating only a partial pathway. Trehalose biosynthesis in all strains relies on the TreYZ (malto-oligosyltrehalose) pathway, as the canonical *otsAB* genes were absent. Notably, only SK18 harbors the complete two-step *gbsAB* system for glycine/betaine synthesis from choline. All three strains carry the constitutive *proABC* genes for proline production, as well as complete glycine cleavage system components and choline transporters, reflecting a versatile arsenal for osmotic and desiccation stress adaptation.

### 2.4. Siderophore Production

**Figure 5:**
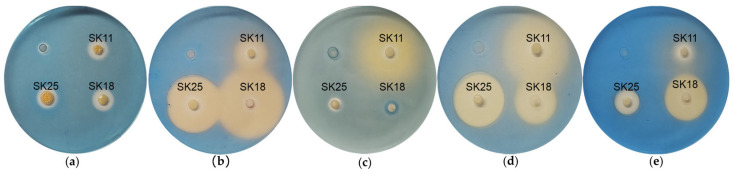
Production of siderophores by lithobiontic actinobacterial strains on standard (**a**) CAS+Fe^3+^ agar, (**b**) CAS+Al^3+^ agar, (**c**) CAS+Co^2+^ agar, (**d**) CAS+Cu^2+^ agar, and (**e**) CAS+Ga^3+^ agar.

Screening on standard CAS agar plates with Fe^3+^ showed siderophore production by all lithobiontic actinobacterial strains (Figure 5). After 6 days of growth, the blue color around colonies changed to yellow, indicating chelation of Fe^3+^ from the CAS complex. Substitution of Fe^3+^ to Al^3+^, Cu^2+^ or Ga^3+^ led to significantly larger halo zones compared to standard conditions for all isolates. Notably, *P. nitroguajacolicus* SK18 formed larger halo zones than *Rhodococcus*-belonging strains (SK11 and SK25).

Genome mining for secondary metabolite biosynthesis gene clusters (BGCs) revealed significant differences in the siderophore biosynthetic potential of the three serpentinite-derived strains (Table 2, Figure S3). *R. oxybenzonivorans* SK11 harbors a large NRP-metallophore/NRPS cluster (cluster 4.3, 58.3 kb) containing a lysine/ornithine N-monooxygenase—a key gene for hydroxamate siderophore biosynthesis [20] with high similarity to rhodochelin, a siderophore previously characterized in *Rhodococcus jostii* RHA1 [21], and low similarity to thermochelin. Additionally, SK11 possesses a distinct aminopolycarboxylic acid cluster (cluster 6.1, 13.4 kb) encoding cysteine synthase, diaminopimelate decarboxylase, and ornithine cyclodeaminase—enzymes involved in amino acid and polyamine metabolism that likely provide precursors for siderophore biosynthesis or function as an independent siderophore system [22].

*R. qingshengii* SK25 contains two NRP-metallophore/NRPS clusters with high similarity to heterobactin B/S2 (cluster 1.2, 57.9 kb; cluster 3.1, 58.2 kb), a catecholate-type siderophore produced by *R. erythropolis* IGTS8 [23]. Notably, cluster 3.1 encodes an MMPL family transporter, providing a dedicated export system for the mature siderophore [24]. Additional clusters with medium and low similarity to erythrochelin and coelichelin were also detected (Table 2).

*P. nitroguajacolicus* SK18 exhibits a distinct siderophore strategy: an NRPS-independent siderophore (NIS) cluster (cluster 2.3, 29.8 kb) with high similarity to desferrioxamine E, containing a lysine/ornithine N-monooxygenase and a pyridoxal-dependent decarboxylase characteristic of hydroxamate siderophore biosynthesis. Interestingly, a lanthipeptide class V cluster (cluster 2.1, 42.1 kb) found in SK18, predicted to synthesize dehydroxynocardamine and regulated via an iron(II)-dependent repressor, suggesting functional coordination between lanthipeptide and siderophore biosynthesis that is rare feature in actinobacteria (Table 2).

### 2.5. Biosurfactant Production

Initial screening on CTAB-methylene blue-agar revealed blue halos around lithobiontic actinobacterial strains after 14 days, indicating extracellular glycolipids or other anionic surfactants (Figure S2).

To evaluate biosurfactant accumulation, lithobiontic actinobacterial strains were grown in five different liquid cultural media: nutrient-rich media LB and Mueller–Hinton broths; a specialized production medium (SPM) with mixed carbon sources; a mineral salts medium (MSM) with defined carbon; and hydrocarbon-amended minimal medium (Table 3). *R. qingshengii* SK25 grown in n-hexadecane mineral medium showed the highest activity, emulsifying dodecane with E_24_ = 100%. *R. oxybenzonivorans* SK11 grown in SPM achieved E_24_ = 83.3%. For *P. nitroguajacolicus* SK18 and *R. oxybenzonivorans* SK11 grown in n-hexadecane mineral medium, E_24_ reached 49.5 and 26.36% on day 5, respectively. No biosurfactant production was observed in LB, Mueller–Hinton broth and MSM under the tested conditions. However, the emulsification assay has limited sensitivity; we cannot exclude the possibility that biosurfactants are produced at concentrations below the detection threshold, especially in LB and Mueller–Hinton broths, where transient foam formation was observed but no stable emulsion was detected.

Genome mining of *R. oxybenzonivorans* SK11 using antiSMASH revealed a vast repertoire of secondary metabolite biosynthetic gene clusters (BGCs). Among these, we identified eight NRPS clusters displaying genetic architectures characteristic of biosurfactant production (Table 4, Figure S3). These clusters range from 41.6 to 69.5 kb and contain features of lipopeptide and glycolipid biosynthesis, including multiple genes involved in fatty acid metabolism (acyl-CoA dehydrogenases, enoyl-CoA hydratases, crotonyl-CoA reductases, short-chain dehydrogenases/reductases) that likely participate in the synthesis and attachment of lipid moieties to peptide backbones [25].

Several clusters exhibit additional features enhancing their biosurfactant potential: cluster 1.2 contains a papA3 acyltransferase gene located close to a glycosyltransferase, a combination characteristic of trehalose ester biosynthesis in *Mycobacterium tuberculosis* [26]; cluster 5.4 encodes an MMPL family transporter, typically dedicated to the export of large lipophilic molecules such as mycobacterial lipids and lipopeptides [24]; and multiple clusters contain glycosyltransferases, suggesting the production of glycosylated derivatives.

Genome mining of *R. qingshengii* SK25 revealed three NRPS clusters with genetic architectures characteristic of biosurfactant production (Table 5). The most promising candidate is cluster 8.1 (42.1 kb), which combines an NRPS core with an acyl-CoA dehydrogenase and acetyl-CoA carboxylase genes involved in fatty acid metabolism, and notably features an MMPL family transporter—a specific export system for large lipophilic molecules in *M. tuberculosis* [24], strongly supporting the production of an exported lipopeptide biosurfactant.

Cluster 5.1 (66.8 kb) contains multiple short-chain dehydrogenase/reductases (SDRs) alongside aldehyde and 3-hydroxyisobutyrate dehydrogenases—enzymes commonly associated with lipid tail modification [25] and three cytochrome P450s suggesting additional oxidative tailoring. Cluster 1.4 (66.6 kb) contains a polyprenol-monophosphomannose synthase positioned next to NAD-dependent epimerases/dehydratases, indicating possible glycosylation of the final product reminiscent of glycopeptide or glycolipid biosynthetic clusters [27].

Genome mining of *P. nitroguajacolicus* SK18 revealed multiple BGCs with genetic features indicative of biosurfactant production (Table 6). Despite possessing only two NRPS clusters (compared to 13–17 in the *Rhodococcus* strains), SK18 harbors a diverse array of PKS and hybrid clusters that may contribute to surface-active compound biosynthesis. However, the most compelling biosurfactant candidate is cluster 4.1 (43.9 kb), an NRPS-like cluster containing crotonyl-CoA reductase, which catalyzes the formation of butyryl-CoA as a starter unit for the biosynthesis of lipid chains in lipopeptides and glycolipids, alongside multiple short-chain dehydrogenase/reductases (SDRs) involved in lipid tail modification [28].

## 3. Discussion

Understanding the adaptive mechanisms of bacteria to lithobiontic environments is fundamental to microbial ecology, as minerals represent some of the most extreme habitats on Earth. These environments are characterized by extreme oligotrophy, desiccation stress, high heavy metal concentrations, and limited nutrient availability, imposing strong selective pressures on colonizing microorganisms [29,30]. In active serpentinization systems, the microbial community is often dominated by well-characterized hydrogen-oxidizing bacteria (e.g., *Serpentinimonas*, *Hydrogenophaga*) and methane-cycling archaea (*Methanobacteriales*, *Methanosarcinales*), which thrive on the geologically produced hydrogen and methane [31].

Weathered serpentinites—where active serpentinization has terminated—represent a distinct and less explored habitat. They constitute a unique lithobiontic niche with low organic carbon content and enhanced levels of heavy metals (e.g., Ni, Cr, Co, Fe), and host specialized microbial communities adapted to these extreme conditions [5].

In this study, we isolated and characterized lithobiontic actinobacteria from serpentinite rock, identifying three strains belonging to the genera *Rhodococcus* and *Paenarthrobacter*: *R. oxybenzonivorans* SK11, *P. nitroguajacolicus* SK18, and *R. qingshengii* SK25. Previously we described endolithic *Rhodococcus* and *Nocardia* strains from other serpentinite environments [32,33,34]; the present study expands the taxonomic diversity of serpentinite-associated actinobacteria and provides new insights into their metabolic versatility.

Phenotypic profiling revealed marked differences in metabolic capacity among the three isolates, reflecting strain-specific strategies for survival in the oligotrophic and desiccated serpentinite environment. These metabolic phenotypes highlight the functional diversity of lithobiontic actinobacterial strains within serpentinites. *R. qingshengii* SK25 showed broad metabolic versatility, a trait mostly advantageous for colonizing lithobiontic environments. In contrast, *R. oxybenzonivorans* SK11 demonstrated a more specialized metabolism, which may reflect adaptation to stable and nutrient-limited conditions. *P. nitroguajacolicus* SK18 occupied an intermediate position, potentially accessing a distinct type of carbon source that reduces niche overlap with the two *Rhodococcus* strains.

Desiccation conditions of weathered serpentinite rocks create a challenge for survival of endolithic microorganisms. In this study, all three lithobiontic actinobacterial strains demonstrated a high desiccation tolerance, maintaining viability at PEG-8000 concentrations as high as 50%. The presence of multiple osmoprotective pathways (ectoine in SK18 and SK25, glycine betaine in SK18, and trehalose/proline in all strains) could represent a key adaptation to the oligotrophic and desiccating environment of weathered serpentinites. Previous studies on xerotolerance in related actinobacteria are in good agreement with our findings. *Arthrobacter* sp. Helios, isolated from a solar panel, was shown to tolerate 35% PEG-6000 by modulating the expression of 324 genes, including enhanced transport of ions, osmoprotectants, biogenic amines, and iron-scavenging systems such as siderophores, indicating a metabolic reprogramming under conditions of reduced water availability [10]. Desiccation at low relative humidity (20%) triggered in the soil strain *R. jostii* RHA1 a global transcriptional response involving upregulation of genes for compatible solute synthesis, protein stabilization via chaperones, and detoxification of reactive oxygen species, allowing the bacterium to survive prolonged drought without sporulation [35]. Thus, we hypothesize that similar adaptation mechanisms to desiccation conditions might be found for our actinobacterial strains; however, subsequent transcriptomic analysis is necessary for confirmation.

Within lithobiontic environments siderophores are required to exchange or acquire essential nutrients. Iron limitation is particularly severe in weathered serpentinites, where high pH and the presence of Fe^3+^ oxides further reduce iron bioavailability [36]. All three lithobiontic actinobacterial strains isolated in this study produced siderophores, as evidenced by clear halo formation on CAS agar, indicating active iron chelation. We found that the substitution of Fe^3+^ with Al^3+^, Cu^2+^, or Ga^3+^ resulted in significantly larger halo zones for all isolates. The broad metal binding specificity likely reflects an adaptive mechanism for heavy metal detoxification along with nutrient acquisition. Siderophore production by bacteria of the genera *Rhodococcus* and *Paenarthrobacter* (formerly *Arthrobacter*) are well established [14,37]. Moreover, members of the *Rhodococcus* have been shown to produce siderophores capable of binding not only iron but also Ga, Al, Cu, and even V, while *Arthrobacter (Paenarthrobacter) aurescens* TC1 similarly forms complexes with multiple metals [38]. Thus, our results align with these findings and further show that the siderophores of serpentinite-isolated strains display differential metal preferences suggesting niche-specific adaptation within the serpentinite rocks.

Along with the physiological capacity of siderophore production, genomic analysis revealed distinct biosynthetic strategies among the lithobiontic actinobacterial strains. Two *Rhodococcus* strains (SK11 and SK25) harbor a broader metabolic repertoire for siderophore biosynthesis, reflecting the well-established metabolic versatility of this genus [39]. Members of the *Rhodococcus* genus are known to encode numerous NRPS clusters involved in siderophore production, including heterobactins, rhodochelin, rhodobactin, rhequichelin and rhequibactin [21,23,40,41,42].

In contrast, *P. nitroguajacolicus* SK18 employs a more specialized nonribosomal peptide synthetase-independent siderophore (NIS) pathway for siderophore biosynthesis leading to desferrioxamine E production together with a unique lanthipeptide-associated cluster. Limited information is available regarding a diversity of siderophores produced by *Paenarthrobacter* strains. Catechol-type siderophores have been described in *Arthrobacter luteolus* suggesting a potential role in rare earth elements binding [37]. Additionally, deferoxamine (or desferrioxamine) produced by *Arthrobacter* sp. QXT-31 has been involved in the extracellular induction of reactive oxygen species (ROS), indicating broader physiological roles beyond iron acquisition [43]. Thus, the lithobiontic actinobacteria use different siderophore biosynthetic strategies reflecting divergent adaptations to iron limitation in the serpentinite environment.

Bacterial metabolites related to biosurfactants have been shown to reduce surface tension to promote surface colonization, biofilm formation, desiccation tolerance, and mineral weathering [44]. Genomic analysis revealed a broad range of biosurfactant biosynthetic gene clusters in all three lithobiontic actinobacterial strains, including NRPS clusters with lipopeptide and glycolipid signatures. Biosurfactant production in these strains is tightly regulated and strongly influenced by the culture medium. The absence of activity in rich media (LB, Mueller–Hinton) and MSM suggests that biosurfactant biosynthesis might be repressed by absence of inducing substrate or nutrient imbalance as previously reported in biosurfactant-producing bacteria of genera *Rhodococcus* and *Arthrobacter (Paenarthrobacter)* [44,45], rather than classical carbon catabolite repression. This is consistent with a bioenergetic trade-off: the cell invests in costly biosurfactant production when growth on water-insoluble substrates requires solubilization and uptake, or under specific nutrient conditions (for example, high C:N ratio) where biosurfactant synthesis provides a selective advantage, possibly by facilitating surface colonization or nutrient acquisition.

Our findings are in good alignment with known mechanisms of surface-active biosynthesis in *Rhodococcus*, particularly, predicted BGCs for glycolipids and lipopeptides, induced on hydrophobic substrates and repressed on rich media, are consistent with data for different *R. erythropolis* strains and *R. ruber* NCIMB 40126, where trehalolipids are synthesized during growth on C11-C16 n-alkanes, vegetable oils, or waste, whereas soluble substrates shift metabolism toward bioflocculants, triacylglycerols (TAGs), and polyhydroxyalkanoates (PHAs) [14]. Similarly, *Arthrobacter (Paenarthrobacter)* species produce lipopeptide biosurfactants such as arthrofactin during growth on glucose or molasses, demonstrating comparable substrate-dependent regulation and surface activity [46]. However, genomic analysis of *Paenarthrobacter* GOM3 isolated from the Gulf of Mexico demonstrated that the strain lacked genes associated with biosurfactant biosynthesis, suggesting strain-specific variability within the genus that may reflect niche adaptation [47]. Thus, the distinct activity profiles among lithobiontic actinobacterial strains may reflect niche differentiation within the heterogeneous serpentinite environment.

## 4. Materials and Methods

### 4.1. Isolation of Bacterial Isolates

The isolates SK11, 18 and 25 were obtained from an aqueous rinsate of crushed serpentine collected from the Aktobe region (Kazakhstan) (48.7797° N, 57.9974° E), which is located within semi-arid climate zone. Isolation was performed by dilution plating on Luria Agar (LA) composed of (g/L): tryptone, 10.0; yeast extract, 5.0; NaCl, 5.0; agar, 20.0). Plates were incubated at 30 °C for 72 h until distinct colonies were isolated.

### 4.2. DNA Extraction and Identification

Genomic DNA from the isolates was extracted using the phenol-chloroform method. A paired-end DNA library was prepared using the NEBNextUltra II DNA Library Prep Kit (Illumina, San Diego, CA, USA) and NEBNext Multiplex Oligos for Illumina (96 Unique Dual Index Primer Pairs) according to manufacturer guidelines. Whole-genome sequencing was carried out by NovaSeq6000 sequencing platform (Illumina) with 2 × 250 bp read length. The quality of raw sequence reads was evaluated by FastQC package (v0.12.1) [48]. The whole genome was assembled using SPAdes (v 4.0.0) software [49].

The genome assembly was evaluated using QUAST (v5.2) program [50]. Genome annotation was performed by RASTtk server, Prokka (v1.14.6) annotation pipeline and the NCBI Prokaryotic Genomes Annotation Pipeline (PGAP) version 5.2 [51]. Pairwise sequence similarities calculated using 16S rRNA genes available via the GGDC web server (http://ggdc.dsmz.de/, accessed on 14 April 2026) [52]. The Average Nucleotide Identity (ANI) of the genomes was analyzed using FastANI v1.34 [53]. Biosynthetic gene clusters were predicted to be present in the genome of strains by the antiSMASH software 8.0 [54].

The whole-genome shotgun projects have been deposited at DDBJ/ENA/GenBank under the accessions JBTGGD000000000 (*Rhodococcus qingshengii* SK25), JBWBIE000000000 (*Rhodococcus oxybenzonivorans* SK11), and JBWBIF000000000 (*Paenarthrobacter nitroguajacolicus* SK18).

### 4.3. Bacterial Identification by MALDI-TOF MS

Colonies of isolates grown overnight on the plates were analyzed using the Bruker Microflex MALDI Biotyper (Bruker Daltonics, Bremen, Germany). Briefly, the whole colonies were directly spotted onto polished steel target plate (MT 96 target) and covered with 1 μL of matrix solution (10 mg α-cyano-4-hydroxycinnamic acid dissolved in 50% acetonitrile, 2.5% trifluoroacetic acid), and air-dried at room temperature. The target plate was then loaded into the Microflex LT instrument, operated in linear positive-ion mode with automated acquisition and analysis via flexControl and MALDI Biotyper 3.1 software. Spectra were processed against the Bruker reference library, with species-level identification assigned for log(score) values ≥2.0, genus-level for ≥1.7, and no reliable identification below 1.7, per manufacturer guidelines. Score values of >1.7 generally indicated relationships at the genus level, and values of >2.0 generally indicated relationships at the species level. The highest score determined final isolate identification.

### 4.4. Phenotypic Analysis

Two Biolog^®^ phenotype microarray types (Biolog Inc., Hayward, CA, USA) were used: GEN III MicroPlates for 94 phenotypic tests (71 carbon source utilization assays and 23 chemical sensitivity assays) and PM5 MicroPlates for biosynthetic pathway/nutrient stimulation assays. All lithobiontic strains were precultured in Luria–Bertani (LB) ((g/L): tryptone, 10.0; yeast extract, 5.0; NaCl, 5.0) broth at 30 °C, 250 rpm, harvested by centrifugation at 5000× *g* for 5 min and washed three times with sterile 0.9% NaCl solution. Final bacterial suspensions of lithobiontic strains were adjusted to an OD600 of 0.1, and 100 μL of aliquots were inoculated into each well. Plates were incubated at 30 °C, 180 rpm, with absorbance measured at 590 nm using a Bio-Rad xMark Microplate Absorbance Spectrophotometer (Bio-Rad, Hercules, CA, USA) over 24–48 h. Data were normalized by subtracting the optical density of the negative control from experimental to yield ΔA_570_ values. All experiments were performed in duplicates.

### 4.5. Xerotolerance Test

To assess desiccation tolerance, bacterial strains (OD_600nm_ = 0.1) were cultivated in Mueller–Hinton medium supplemented with increasing concentrations (10–50% *w*/*v*) of polyethylene glycol (PEG) 8000. Bacterial cultures were incubated at 30 °C, 250 rpm for 7 days. Growth was monitored daily by measuring A_600nm_ following determination of viability by plating serial dilutions and counting colony-forming units (CFU/mL). All experiments were performed in duplicate.

### 4.6. Screening for the Production of Siderophores

Bacterial strains were screened for siderophores production using the Chrome Azurol S (CAS) assay method [55]. Briefly, the CAS blue dye solution was prepared by slowly adding 2 mM of CAS and 1 mM FeCl_3_ × 6H_2_O in 10 mM HCl to a solution of 73 mg hexadecyl trimethylammonium bromide (HDTMA) in 40 mL distilled water, resulting in deep-blue-colored solution. To assess the metal-binding ability of the siderophores, alternative CAS solutions were prepared by substituting Fe^3+^ with 1 mM stocks of other metal salts (CoCl_2_, AlCl_3_, CuSO_4_, and GaCl_3_) in 10 mM HCl. The final CAS solutions were autoclaved separately. The basal medium containing MM9, g/L (KH_2_PO_4_ (0.3), NaCl (0.5), NH_4_Cl (1)), PIPES (30.24 g/L) and agarose (1 g/L) was autoclaved separately. Desferrated casamino acids (3 g/L) were prepared separately and sterilized by filtration through a 0.45 µm filter. All components were then aseptically mixed and poured into plastic Petri plates. LB-precultured overnight bacterial isolates were harvested by centrifugation at 5000× *g* for 5 min, washed three times with sterile 0.9% NaCl, resuspended in the 0.9% NaCl to OD_600nm_ = 1.0. A 5 µL bacterial suspension was spotted onto the CAS agar plates. Plates were incubated at 30 °C for 7 days.

### 4.7. Screening for the Production of Biosurfactant

#### 4.7.1. Blue-Agar Plate Method

Bacterial isolates were screened using the CTAB-methylene blue-agar plate assay for detection of anionic biosurfactants [56]. LB-precultured overnight bacterial isolates were harvested by centrifugation at 5000× *g* for 5 min, washed three times with sterile 0.9% NaCl, and resuspended in the 0.9% NaCl to OD_600nm_ = 1.0. A 5 µL bacterial suspension was spotted onto the agar plates. Plates were incubated at 30 °C for 14–21 days.

#### 4.7.2. Biosurfactant Production in Liquid Media

Bacterial strains were grown at 30 °C, 150 rpm for 18 h in LB broth. Cells were harvested by centrifugation at 10,000× *g* for 5 min, washed three times with sterile 0.9% NaCl. Bacterial cells were inoculated (OD_600nm_ = 0.1) into the test media for optimization of biosurfactants production. The medium compositions were: 1. LB broth; 2. Mueller–Hinton broth; 3. Modified Production Medium: ((g/L): K_2_HPO_4,_ 1.0, MnSO_4_·7H_2_O, 0.01, yeast extract, 0.1, glucose, 10.0, glycerol, 10.0, NaNO_3,_ 1.0) [57]; 4. Modified mineral salt medium (MSM): ((g/L): KH_2_PO_4_, 1.2; K_2_HPO_4_, 4.8; MgSO_4_·7H_2_O, 0.25; FeSO_4_ × 7H_2_O, 0.0025; CaCl_2_·2H_2_O, 0.026; NaCl, 5.0; NH_4_NO_3_, 1.0; glucose, 20.0); 5. M9 + 1% hexadecane: ((g/L): Na_2_HPO_4,_ 6.77, KH_2_PO_4,_ 3.0, NH_4_Cl, 1.0, NaCl, 0.5, stock solutions of MgSO_4_·7H_2_O and CaCl_2_ were added to final concentration 246 and 100 μM, respectively). The C:N ratio was calculated as the molar ratio of total elemental carbon to total elemental nitrogen per liter of medium.

Cultures were incubated at 30 °C, 150 rpm for 14 days. The emulsification index (E_24_) was monitored on days 5, 10, and 14. Briefly, the cell-free supernatant was mixed with an equal volume of dodecane, vortexed vigorously for 2 min, and incubated for 24 h at room temperature to allow for phase separation. All experiments were performed in duplicate. The E_24_ was calculated as follows [58]:

$$E_{24}=\left(\frac{height\;of\;the\;emulsified\;layer\;(mm)}{height\;of\;the\;total\;upper\;phase\;(mm)}\right)\times 100\%$$

## 5. Conclusions

All our results suggest that lithobiontic actinobacteria from weathered serpentinites rely on an extracellular molecular repertoire that enable adaptation under oligotrophy, desiccation, and metal stress. The combination of broad metabolic flexibility, osmoprotectant accumulation, siderophore production, and biosurfactant activity supports not only survival within the rock matrix but also active interaction with mineral surfaces and acquisition of limiting nutrients in this extreme geochemical environment. These traits are consistent with the view that serpentinite-hosted endoliths are not passive inhabitants of mineral niches, but active participants in microbe–mineral interactions that may contribute to weathering, element mobilization, and the progressive development of microenvironments. In this context, xerotolerant Actinobacteria belonging to *Rhodococcus* and *Paenarthrobacter* genera emerge as promising models for understanding how heterotrophic bacteria colonize and function in weathered ultramafic rocks, and for identifying microbial mechanisms in the stress tolerance and mineral transformation. Beyond their ecological significance, these traits may also provide a basis for agricultural applications, particularly for improving plant resilience in infertile and arid environments.

## Figures and Tables

**Table 1:** Genetic potential for compatible solute biosynthesis and desiccation tolerance in lithobiontic actinobacterial strains.

Compatible Solute/Pathway	Key Enzymes/Markers	*R. oxybenzonivorans* SK11	*P. nitroguajacolicus* SK18	*R. qingshengii* SK25
Ectoine	*ectABC* (L-2,4-diaminobutyrate pathway)	Partial (kinase, dehydrogenase)	Complete (*ectABC*)	Complete (*ectABC*)
Trehalose	*otsAB* (TPS/TPP)	–	–	–
	TreYZ (malto-oligosyltrehalose pathway)	Complete	Complete	Complete
	*treS* (trehalose synthase)	–	–	–
Glycine betaine	*gbsAB* (choline dehydrogenase/betaine aldehyde dehydrogenase)	–	Complete	–
	Aldehyde dehydrogenase (alternative)	+	–	–
Proline	*proABC* (glutamate → proline)	Complete	Complete	Complete
Choline transport	*betT*/BCCT family	+	+	+
Glycine cleavage system	GCV complex	Complete	Complete	Complete

Complete indicates presence of all genes required for the canonical biosynthesis pathway. Partial indicates presence of some but not all key genes. – indicates not detected in the genomes.

**Table 2:** NRPS biosynthetic gene clusters identified in the genomes of lithobiontic actinobacterial strains with predicted involvement in siderophores synthesis.

Strain	Cluster	Type	Size (bp)	Similarity to Known Siderophores	Key Biosynthetic Genes	Additional Features
*R. oxybenzonivorans* SK11	4.3	NRP-metallophore + NRPS	58,268	Rhodochelin (high similarity); thermochelin (low similarity)	Lysine/ornithine N-monooxygenase; two acyl-CoA dehydrogenases; two SDRs *; two AMP-dependent synthetases	Two enoyl-CoA hydratases; NAD-dependent epimerase/dehydratase; iron(II)-dependent regulator
	6.1	Aminopolycarboxylic-acid	13,449	Putative siderophore-related (aminopolycarboxylate type)	Cysteine synthase; diaminopimelate decarboxylase; ornithine cyclodeaminase	Involved in amino acid/polyamine metabolism—may contribute precursor biosynthesis for siderophores
*R. qingshengii* SK25	1.2	NRP-metallophore + NRPS	57,935	Heterobactin B/S2 (high similarity); erythrochelin (medium); coelichelin (low)	Isochorismatase; isochorismate synthase; two AMP-dependent synthetases; SDR	Methyltransferase; catecholate-type siderophore machinery
	3.1	NRP-metallophore + NRPS	58,192	Heterobactin-like (high similarity)	Lysine/ornithine N-monooxygenase; AMP-dependent synthetase; methyltransferase; formyltransferase	MMPL family transporter—dedicated export system;
*P. nitroguajacolicus* SK18	2.3	NI-siderophore	29,842	Desferrioxamine E (high similarity)	Lysine/ornithine N-monooxygenase; SDR; aminotransferase; pyridoxal-dependent decarboxylase	Amidohydrolase; sugar-binding lipoprotein—possible transport
	2.1	Lanthipeptide-class-v	42,081	Novel lanthipeptide (co-occurring with siderophore-related NRPS)	Oxidoreductase; mandelate racemase; APH domain	Regulated via iron(II)-dependent repressor; predicted to synthesize dehydroxynocardamine in conjunction with siderophore system

* SDR—short-chain dehydrogenase/reductase; NI-siderophore—NRPS-independent siderophore; NRP-metallophore—nonribosomal peptide metallophore; RRE—RiPP recognition element; MMPL—mycobacterial membrane protein large.

**Table 3:** Biosurfactant emulsification activity (E_24_) of lithobiontic actinobacterial strains in cultural supernatants.

Media	SPM + Mixed Carbon Sources	M9 + 1% Hexadecane
C:N ratio, (mol/mol)	56.1:1	37.8:1
Strain		
*R. oxybenzonivorans* SK11	83.3 ± 0	49.95 ± 23.5
*P. nitroguajacolicus* SK18	nd	26.36 ± 12.0
*R. qingshengii* SK25	31.25 ± 8.8	100 ± 0
Negative control *	nd	nd
Positive control ^§^	100	

* Blank medium (no activity); ^§^ 1% Tween 80; nd, not detected. Data represent mean ± SD from two independent experiments.

**Table 4:** NRPS biosynthetic gene clusters identified in the genome of *Rhodococcus oxybenzonivorans* SK11 with predicted involvement in biosurfactant production.

Cluster	Size (bp)	Predicted Product Type	Key Tailoring Genes Associated with Lipid Metabolism	Additional Features
1.1 NRPS	69,511	Lipopeptide biosurfactant	Crotonyl-CoA reductase; three SDRs	Polyprenol-monophosphomannose synthase—potential glycosylation
1.2 NRPS	66,814	Glycolipid (trehalose ester/mycobactin-like)	PapA3 acyltransferase; SDR	Glycosyltransferase
2.1 NRPS	66,688	Lipopeptide biosurfactant	Two acyl-CoA dehydrogenases; 3-hydroxybutyryl-CoA dehydrogenase; aldehyde dehydrogenase	Condensation domain-containing protein
2.2 NRPS	66,682	Lipopeptide biosurfactant	Crotonyl-CoA reductase; three α/β hydrolases	AMP-dependent synthetase/ligase
2.5 NRPS	62,245	Glycosylated peptide (glycolipid candidate)	Two nucleotide-sugar dehydrogenases; acyltransferase 3	Sugar transferase
3.1 NRPS	66,023	Lipopeptide biosurfactant	Acyl-CoA dehydrogenase; 3-hydroxybutyryl-CoA dehydrogenase; ketoacyl synthase	Two condensation domains
5.2 NRPS	44,458	Modified NRPS product	Alcohol dehydrogenase; aldehyde dehydrogenase; SDR	Cytochrome P450
5.4 NRPS	41,65_	Lipopeptide biosurfactant	Crotonyl-CoA reductase; two acyl-CoA dehydrogenases; two SDRs; enoyl-CoA hydratase	MMPL family transporter—dedicated export system

**Table 5:** NRPS biosynthetic gene clusters identified in the genome of *Rhodococcus qingshengii* SK25 with predicted involvement in biosurfactant production.

Cluster	Size (bp)	Predicted Product Type	Key Tailoring Genes Associated with Lipid Metabolism	Additional Features
1.4 NRPS, RRE-containing *	66,577	Lipopeptide/glycolipid candidate	NAD-dependent epimerase/dehydratase—sugar/lipid modification	Polyprenol-monophosphomannose synthase—glycosylation; thioesterase
5.1 NRPS	66,793	Lipopeptide biosurfactant candidate	Three SDRs; aldehyde dehydrogenase; 3-hydroxyisobutyrate dehydrogenase	Three cytochrome P450s; AMP-dependent synthetase
8.1 NRPS	42,125	Lipopeptide biosurfactant candidate	Acyl-CoA dehydrogenase; acetyl-CoA carboxylase complex	MMPL family transporter—dedicated export system; AMP-dependent synthetase

* RRE-containing—RiPP Recognition Element.

**Table 6:** NRPS biosynthetic gene clusters identified in the genome of *P. nitroguajacolicus* SK18 with predicted involvement in biosurfactant production.

Cluster	Size (bp)	Predicted Product Type	Key Tailoring Genes Associated with Lipid Metabolism	Additional Features
4.1 NRPS-like	43,891	Lipopeptide biosurfactant candidate	Crotonyl-CoA reductase; two SDRs	Phenylalanine-specific permease—amino acid transport; GCN5 acetyltransferase; alpha/beta hydrolase

## Data Availability

The original contributions presented in this study are included in the article/Supplementary Material. Further inquiries can be directed to the corresponding author.

## Supplementary Materials

The following supporting information can be downloaded at: https://www.mdpi.com/article/10.3390/ijms27104233/s1.

## Abbreviations

The following abbreviations are used in this manuscript:

dDDH	DNA–DNA hybridization
ANI	Average nucleotide identity
CAS	Chrome Azurol S
HDTMA	Hexadecyl trimethylammonium bromide
PEG	Polyethylene glycol
NRP	Nonribosomal peptide
NRPS	Nonribosomal peptide synthetase
LA	Luria Agar
LB	Luria Broth
MSM	Mineral salts medium
SPM	Specialized production medium
MALDI-TOF MS	Matrix-Assisted Laser Desorption/Ionization—Time of Flight Mass Spectrometry
GBDP	Genome BLAST distance phylogeny
CFU	Colony-forming units
BGCs	Biosynthetic gene clusters
NIS	NRPS-independent siderophore
CTAB	Cetryltrimethylammonium bromide
SDRs	Short-chain dehydrogenase/reductases
TAGs	Triacylglycerols
