# Supplementary material for: Extracellular Molecular Repertoire of Xerotolerant Actinobacteria Colonizing Serpentinite Rocks
Source: Int J Mol Sci. 2026 May 9;27(10):4233. doi: 10.3390/ijms27104233 (PMC13206832; doi:10.3390/ijms27104233)
Supplement: Supplementary file 1 [file ijms-27-04233-s001.zip › ijms-4292193-supplementary.pdf]

Supplementary Information

Extracellular Molecular Repertoire of Xerotolerant Actinobacteria Colonizing Serpentinite Rocks

A.A. Elistratova[1], E.N. Dekhanova[1], D. Kamaldinova, E.I. Shagimardanova[2,3], M.R. Sharipova[1], M.F. Cohen[4], I.V. Khilyas[1]*

[1]Institute of Fundamental Medicine and Biology, Kazan (Volga Region) Federal University, Kazan, Russian Federation.

[2] Genomic and Bioimaging Core Facility, Moscow, Russian Federation.

[3] Life Improvement by Future Technologies (LIFT) Center, Moscow, Russian Federation.

[4] Laboratory of Multiomics Technologies of Living Systems, Institute Fundamental Medicine and Biology, Kazan (Volga Region) Federal University, Kazan, Russian Federation.

[5] University of California Cooperative Extension, Santa Clara County, San Jose, California, USA.

*Corresponding author: irina.khilyas@gmail.com

Table S1. Identification of lithobiontic isolates by MALDI-TOF MS (Biotyper).

Isolate	Best MALDI-TOF MS identification (score)
SK11	*Rhodococcus imtechensis* (1.51)
SK18	*Arthrobacter nitroguajacolicus* (2.02)
SK25	*Rhodococcus erythropolis* (1.81)

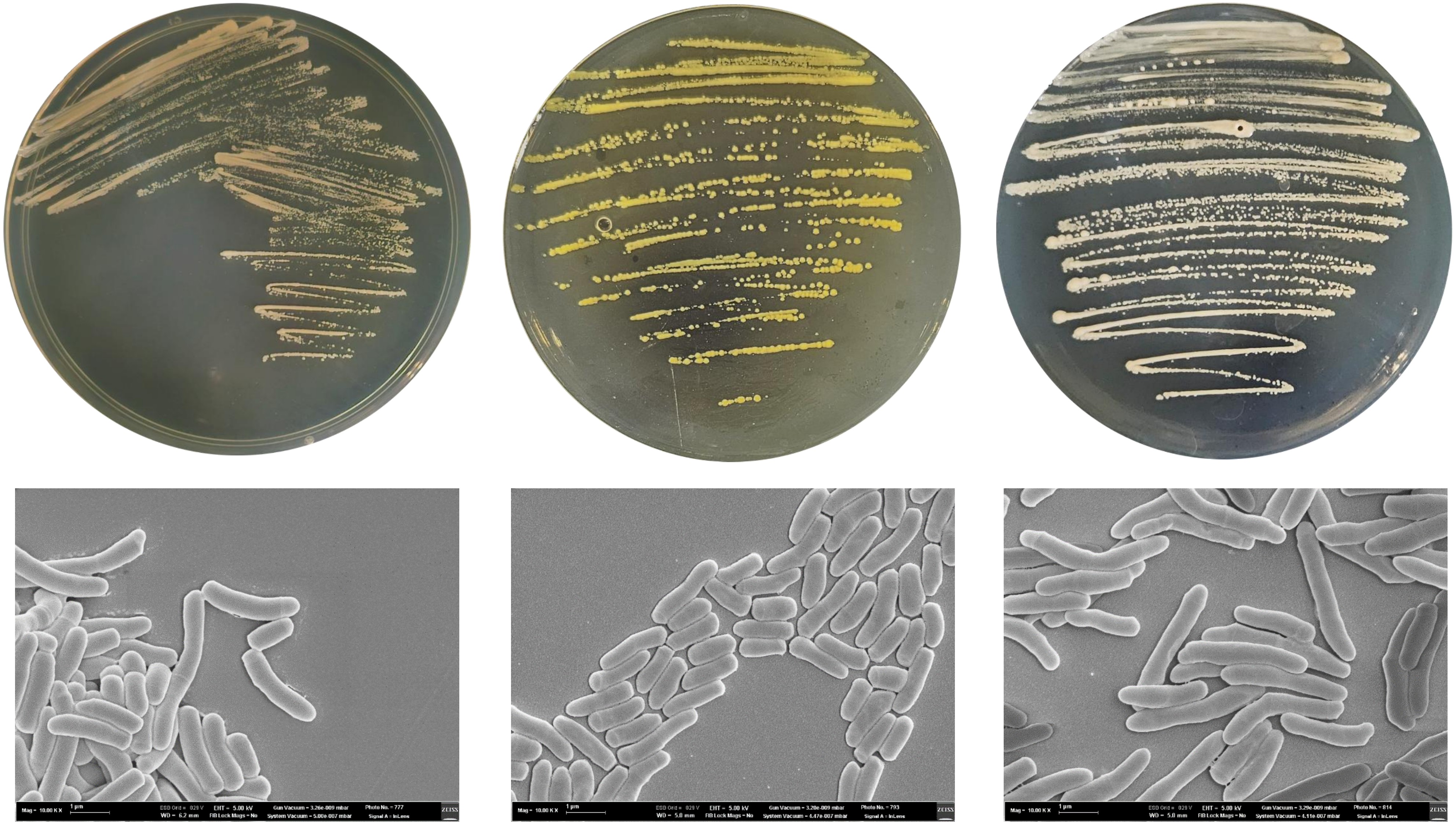

Rhodococcus oxybenzonivorans SK11   *Paenarthrobacter nitroguajacolicus* SK18   *Rhodococcus qingshengii* SK25

Figure S1. Lithobiontic actinobacterial strains grown on LB agar after 24 h of incubation at 30 °C. The streak plating technique was used to obtain isolated colonies. Scanning electron micrograph of three lithobiontic actinobacterial strains grown on LB agar.

Table S2. The general genome characteristics of the lithobiontic strains obtained after annotation using PGAP* and RAST**.

Isolates	SK11		SK18		SK25	
Annotation	PGAP	RAST	PGAP	RAST	PGAP	RAST
Genome size, bp	7,807,245	7,821,393	4,761,278	4,762,147	6,885,859	6,886,367
Total number of genes	7,049	7875	4,497	4645	6,493	6867
Protein coding genes	7,049	7818	4,410	4584	6,380	6809
Number of RNAs	61	57	64	61	66	58
Contigs	85	104	24	25	46	47
N50	514730		456959		422773	
GC%	65		62		62	

* PGAP - NCBI Prokaryotic Genomes Annotation Pipeline
** RAST - Rapid Annotation using Subsystem Technology

Table S3. Pairwise comparisons of *Rhodococcus oxybenzonivorans* SK11 genomes vs. *Rhodococcus* strain genomes.

Query strain	Subject strain	dDDH (d0. in %)	C.I. (d0. in %)	dDDH (d4. in %)	C.I. (d4. in %)	dDDH (d6. in %)	C.I. (d6. in %)	G+C content difference (in %)
SK11	*Rhodococcus oxybenzonivorans* S2-17	57.1	[53.5 - 60.6]	72.4	[69.4 - 75.3]	60.7	[57.5 - 63.9]	0.35
SK11	*Rhodococcus tibetensis* FXJ9.536	49.6	[46.2 - 53.0]	39.2	[36.7 - 41.7]	47.1	[44.1 - 50.1]	0.62
SK11	*Rhodococcus mengyinensis* NM-2T	32.4	[29.0 - 35.9]	27.1	[24.7 - 29.6]	30.1	[27.2 - 33.2]	1.95

Table S4. Pairwise comparisons of *Paenarthrobacter nitroguajacolicus* SK18 genomes vs. *Paenarthrobacter* strain genomes.

Query strain	Subject strain	dDDH (d0. in %)	C.I. (d0. in %)	dDDH (d4. in %)	C.I. (d4. in %)	dDDH (d6. in %)	C.I. (d6. in %)	G+C content difference (in %)
SK18	*Paenarthrobacter nitroguajacolicus* JCM 14115	79.7	[75.8 - 83.2]	79.1	[76.1 - 81.8]	82.5	[79.2 - 85.4]	0.28
SK18	*Paenarthrobacter aurescens* NBRC 12136	62.8	[59.0 - 66.4]	29.5	[27.1 - 32.0]	52.8	[49.7 - 55.9]	0.08
SK18	*Paenarthrobacter histidinolovorans* JCM 2520	47.6	[44.2 - 51.0]	25.6	[23.2 - 28.0]	40.5	[37.5 - 43.5]	0.95

Table S5. Pairwise comparisons of *Rhodococcus qingshengii* SK18 genomes vs. *Rhodococcus* strain genomes.

Query strain	Subject strain	dDDH (d0. in %)	C.I. (d0. in %)	dDDH (d4. in %)	C.I. (d4. in %)	dDDH (d6. in %)	C.I. (d6. in %)	G+C content difference (in %)
SK25	*Rhodococcus jialingiae* djl-6-2	84.1	[80.3 - 87.2]	88.6	[86.2 - 90.7]	87.7	[84.7 - 90.2]	0.07
SK25	*Rhodococcus enclensis* NIO-1009	75.3	[71.3 - 78.9]	87.4	[84.8 - 89.5]	80.1	[76.7 - 83.1]	0.04
SK25	*Rhodococcus qingshengii* JCM 15477	76.7	[72.7 - 80.3]	87.4	[84.8 - 89.5]	81.4	[78.0 - 84.3]	0.02

Table S6. Detailed list of genes associated with compatible solute biosynthesis and desiccation tolerance in lithobiontic actinobacterial strains.

Strain	Compatible solute	Gene / Protein	Accession	Predicted function
R. oxybenzonivorans SK11	Ectoine	Aspartate kinase	MHN1728214.1	Lysine biosynthesis / precursor for ectoine
		Aspartate-semialdehyde dehydrogenase	MHN1728215.1	Precursor biosynthesis
		Diaminobutyrate-2-oxoglutarate transaminase	MHN1728600.1	Ectoine pathway (EctB)
		Diaminobutyrate acetyltransferase	MHN1728599.1	Ectoine pathway (EctA)
		Ectoine synthase	MHN1728601.1	Ectoine pathway (EctC)
		Glycogen debranching protein	MHN1727883.1	Trehalose biosynthesis (TreZ)
	Trehalose	Glucose-1-phosphate adenylyltransferase	MHN1725198.1	Glycogen synthesis
		1,4-alpha-glucan branching protein	MHN1730175.1	Glycogen branching
		Glycogen debranching protein	MHN1727883.1	TreYZ pathway
		Malto-oligosyltrehalose synthase	MHN1728373.1	TreY
		Malto-oligosyltrehalose trehalohydrolase	MHN1728382.1	TreZ
	Proline	Glutamate 5-kinase	MHN1728604.1	ProB
		Glutamate-5-semialdehyde dehydrogenase	MHN1728594.1	ProA
		Pyrroline-5-carboxylate reductase	MHN1726524.1	ProC
P. nitroguajacolicus SK18	Ectoine	Aspartate kinase	MHN1803889.1	Precursor biosynthesis
		Aspartate-semialdehyde dehydrogenase	MHN1803216.1	Precursor biosynthesis
		Diaminobutyrate-2-oxoglutarate transaminase	MHN1803217.1	EctB
		Diaminobutyrate acetyltransferase	MHN1803218.1	EctA
		Ectoine synthase	MHN1803219.1	EctC
	Trehalose	Glucose-1-phosphate adenylyltransferase	MHN1804822.1	Glycogen synthesis
		1,4-alpha-glucan branching enzyme	MHN1807177.1	Glycogen branching

		Glycogen debranching protein	MHN1803309.1, MHN1807187.1	TreYZ pathway
		Malto-oligosyltrehalose synthase	MHN1803310.1	TreY
		Malto-oligosyltrehalose trehalohydrolase	MHN1803311.1	TreZ
	Glycine betaine	GMC family oxidoreductase	MHN1804039.1	Choline dehydrogenase (GbsB)
		Gamma-aminobutyraldehyde dehydrogenase	MHN1803679.1	Betaine aldehyde dehydrogenase (GbsA)
		Aldehyde dehydrogenase family protein	MHN1804038.1	Alternative aldehyde dehydrogenase
	Proline	Glutamate 5-kinase	MHN1806142.1	ProB
		Glutamate-5-semialdehyde dehydrogenase	MHN1806141.1	ProA
		Pyrroline-5-carboxylate reductase	MHN1802853.1	ProC
R. qingshengii SK25	Ectoine	Aspartate kinase	MHL7913027.1	Precursor biosynthesis
		Aspartate-semialdehyde dehydrogenase	MHL7913028.1	Precursor biosynthesis
		Diaminobutyrate-2-oxoglutarate transaminase	MHL7914824.1	EctB
		Diaminobutyrate acetyltransferase	MHL7914823.1	EctA
		Ectoine synthase	MHL7914825.1	EctC
		Phytanoyl-CoA dioxygenase family protein	MHL7915158.1	Possible hydroxyectoine (EctD)
	Trehalose	Glucose-1-phosphate adenylyltransferase	MHL7911060.1	Glycogen synthesis
		1,4-alpha-glucan branching protein	MHL7914944.1	Glycogen branching
		Glycogen debranching protein	MHL7913220.1	TreYZ pathway
		Malto-oligosyltrehalose synthase	MHL7913221.1	TreY
		Malto-oligosyltrehalose trehalohydrolase	MHL7913239.1	TreZ
	Proline	Glutamate 5-kinase	MHL7914829.1	ProB
		Glutamate-5-semialdehyde dehydrogenase	MHL7914817.1	ProA
		Pyrroline-5-carboxylate reductase	MHL7916166.1	ProC

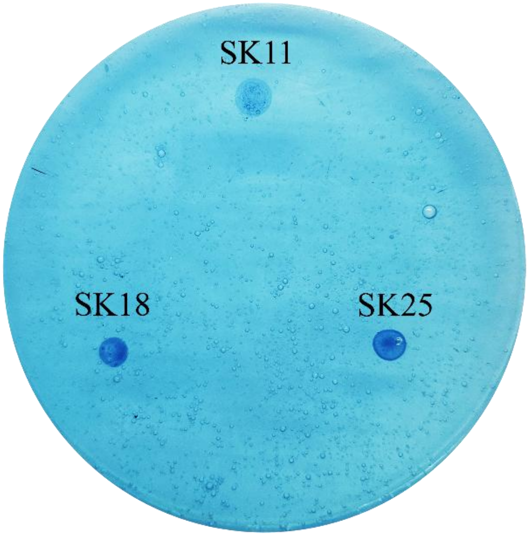

Figure S2. Screening for extracellular glycolipids and other anionic surfactants produced by lithobiontic actinobacterial strains on CTAB-methylene blue agar. The formation of dark blue color around colonies indicates the production of anionic surfactants, which form insoluble ion pairs with the cationic detergent cetyltrimethylammonium bromide (CTAB) and methylene blue.

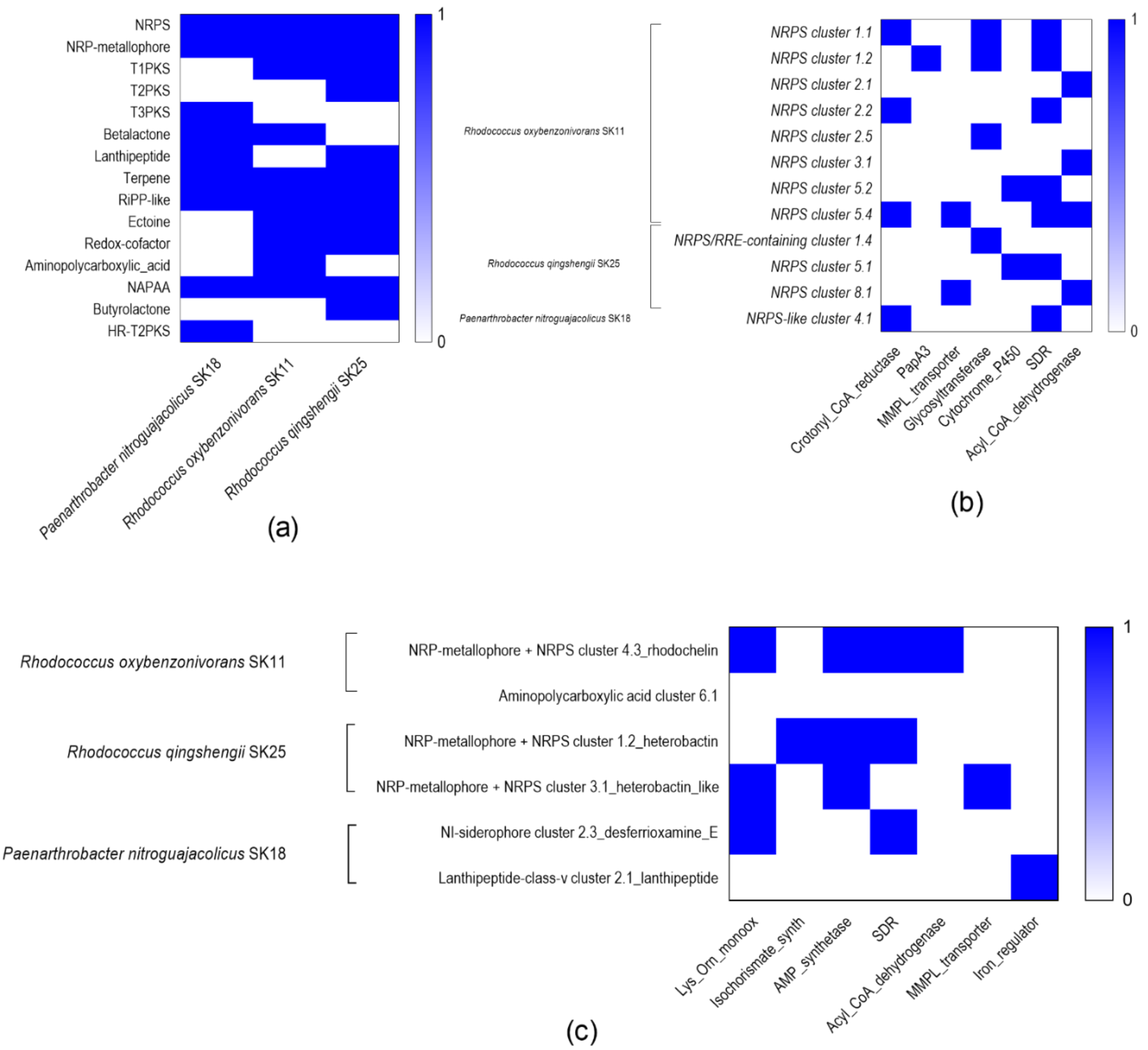

Figure S3. (a) Binary heat map illustrating the distribution of biosynthetic gene cluster categories among three lithobiontic actinobacterial strains. (b) Binary heat map illustrating the presence or absence of key enzymatic markers in NRPS clusters predicted to be involved in biosurfactant production. (c) Binary heat map of siderophore-associated biosynthetic gene clusters and their key enzymatic markers. Color indicates presence (blue) or absence (white) of each feature.
